# An International, Web-Based, Prospective Cohort Study to Determine Whether the Use of ACE Inhibitors prior to the Onset of Scleroderma Renal Crisis Is Associated with Worse Outcomes—Methodology and Preliminary Results

**DOI:** 10.1155/2010/347402

**Published:** 2010-09-14

**Authors:** Marie Hudson, Murray Baron, Ernest Lo, Joanna Weinfeld, Daniel E. Furst, Dinesh Khanna

**Affiliations:** ^1^Jewish General Hospital and McGill University, Montreal, QC, Canada H3T 1E2; ^2^Geffen School of Medicine, University of California, Los Angeles, LA 90095, USA

## Abstract

*Background*. To describe the methodology of a study designed to determine whether systemic sclerosis (SSc) patients with incident scleroderma renal crisis (SRC) on angiotensin converting enzyme (ACE) inhibitors prior to the onset of SRC have worse outcomes. *Methods*. Prospective, international cohort study of SRC subjects identified through an ongoing web-based survey. Every second Friday afternoon, an e-mail was sent to 589 participating physicians to identify new cases of SRC. Death or dialysis at one year after the onset of SRC will be compared in patients exposed or not to ACE inhibitors prior to the onset of SRC. *Results*. Fifteen months after the start of the survey, we had identified 76 incident cases of SRC. Of these, 66 (87%) had a hypertensive SRC and 10 (13%) a normotensive SRC. Twenty-two percent (22%) of the patients were on an ACE inhibitor immediately prior to the onset of the SRC. To date, we have collected one-year follow-up data on approximately 1/3 of the cohort. Of these, over 50% have died or remain on dialysis at one year. *Conclusion*. An international, web-based cohort study design is a feasible method of recruiting a substantial number of patients to study an infrequent vascular manifestation of SSc.

## 1. Introduction

Scleroderma renal crisis is an infrequent but life-threatening complication of systemic sclerosis (SSc) [1]. It was previously associated with significant morbidity, including chronic renal failure and dialysis, and high mortality. However, since the advent of angiotensin converting enzyme (ACE) inhibitors, the outcome of SRC has improved dramatically [2]. There is also a perception among experts that the incidence of SRC has fallen over the past years. This is thought to be due in part to the more liberal use of ACE inhibitors to treat Raynaud's phenomenon and hypertension in SSc [3]. 

Given the benefits of ACE inhibitors in SRC and the perceived decrease in incidence in SRC, some experts have advocated the use of prophylactic ACE inhibitors even in the absence of Raynaud's or hypertension [3]. However, others have argued that there is no clear rationale for this since it has been demonstrated that most SSc patients do not have hyper-reninemia prior to the onset of SRC [4]. In addition, recent retrospective data in patients with SRC suggested that ACE inhibitors prior to the onset of SRC may have worse outcomes than those not taking these drugs [5–7]. This has been hypothesized to be due to the fact that those on ACE inhibitors may have normotensive SRC and diagnosis may thus be delayed in these patients.

Given the belief that the incidence of SRC seems to have fallen over the past years due to the increasing use of ACE inhibitors, some experts have proposed undertaking a large, simple randomized trial to confirm this finding [8]. However, concerns based on the preliminary data that suggests that patients taking ACE inhibitors who develop SRC may have worse outcomes remain. Thus, prior to undertaking a large, randomized trial, we believed that there was a real need to obtain additional data to assess whether in fact the use of ACE inhibitors prior to the onset of SRC was associated with worse outcomes.

We therefore undertook a study to determine whether SSc patients with incident SRC on ACE inhibitors immediately prior to the onset of SRC have worse outcomes, defined as dialysis dependence or death after one year than those not on these drugs prior to the onset of SRC. The purpose of this paper is to describe the methodology and preliminary data of this study. In particular, we wish to highlight the advantages and disadvantages associated with using survey methodology via the internet to study uncommon vascular manifestations of SSc.

## 2. Methods

### 2.1. Design

Prospective, international cohort study of subjects identified through an ongoing web-based survey.

### 2.2. Study Subjects

In September 2008, we compiled an e-mail list of physicians with an interest in SSc from the Canadian Scleroderma Research Group, the Scleroderma Clinical Trials Consortium, the EULAR Scleroderma Trials and Research (EUSTAR) group, and other international collaborators, in particular from Colombia, Mexico, and Australia. They were contacted and invited to participate in the web-based survey. Thereafter, 589 participating physicians were sent an e-mail every second Friday afternoon asking them simply: “Have you diagnosed a case of SRC in the past two weeks”. They were asked to check a yes/no box. If the answer was no, and in most cases it was, then that was all that was required of them for that period. If the answer was yes, they were then asked to answer a simple, short survey about their case requiring about 5 minutes to complete. The survey was developed and conducted using SurveyMonkey, a simple, inexpensive, web-based survey tool. We initially intended to collect cases over a 52-week period, but since we were still identifying new cases at the end of that period, we chose to continue the survey beyond that point.

### 2.3. Definition of SRC

For the purposes of the study, a patient was diagnosed with SRC if he/she was *diagnosed with SRC by the recruiting physician*. We nevertheless collected data on the signs and symptoms that the physicians relied on to make their diagnosis (Box 1).

### 2.4. Covariates

The survey allowed us to collect data on the following variables:

1. patient demographics (age, sex, race/ethnicity),
2. disease characteristics (limited versus diffuse, disease duration, autoantibodies),
3. blood pressure and renal function prior to the onset of the SRC,
4. current use of ACE inhibitor or ARB immediately prior to SRC onset, and if so, reasons for such use (Raynaud's, hypertension, prophylaxis because of concurrent corticosteroid use, simple prophylaxis); name of drug, current dose,
5. concomitant medications, including glucocorticoids, cyclosporine and nonsteroidal anti-inflammatories,
6. signs and symptoms used to diagnose SRC.

### 2.5. Outcomes

The primary outcomes of interest were defined as death or dialysis one year after the onset of SRC. Secondary outcomes included renal function after one year. One year after a patient was identified, a simple follow-up case report form is sent to the recruiting physician. At study completion, the rates of dialysis or death after one year in SSc patients with SRC exposed to ACE inhibitors at the time when they developed SRC will be compared to the rates in those not on ACE inhibitors at the time they developed their SRC.

### 2.6. Sample Size Considerations

The main objective of this study was to determine whether there was harm associated with using ACE inhibitors prior to the onset of SRC. In computing an estimated sample size, we made the following assumptions: (1) estimated prevalence of ACE inhibitor exposure prior to the onset of SRC approximately 25% (ratio of exposed to non-exposed 1 : 3); (2) prevalence of death or dialysis after one year in the nonexposed of approximately 50% [2]; (3) risk of death or dialysis associated with exposure approximately twofold [5], and (4) loss to follow-up of about 10%. We thus calculated that a total sample of approximately 60 subjects would be needed to have 80% power to detect the estimated increased risk in poor outcomes in those exposed compared to those unexposed to ACE inhibitors.

### 2.7. Ethical Considerations

Central research ethics approval was obtained for this study from the ethics review board of the Jewish General Hospital, Montreal, Canada. Some recruiting physicians also sought local ethics approval prior to enrolling patients into the survey.

## 3. Results

**Figure 1:**
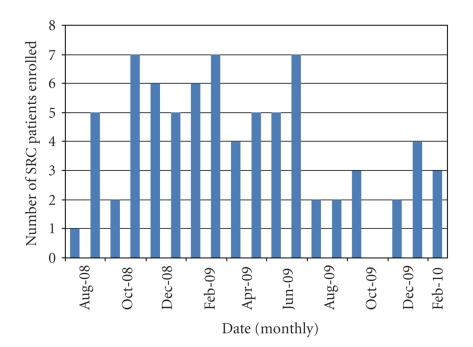
Rate of recruitment of study subjects.

**Figure 2:**
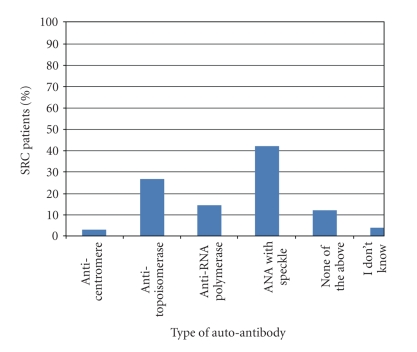
Autoantibodies.

As of February 2010, fifteen months after the start of the survey, we had identified 76 incident cases of SRC (Figure 1). Mean age of the cohort was 53 (±12 years), 68% were women, 72% were White, 68% had diffuse SSc, and median disease duration since the onset of the first non-Raynaud's symptom was 1.5 years (Table 1). Approximately half of the cases were from Canada and the United States, and the other half from elsewhere around the world. Fifteen percent (15%) of patients were positive for an RNA polymerase autoantibody (although not all centers tested for this antibody) and 42% for a speckled antinuclear antibody (Figure 2).

Of the 76 patients, 66 (87%) had a hypertensive SRC and 10 (13%) a normotensive SRC according to the recruiting physician. Twenty-two percent (22%) of the patients were on an ACE inhibitor and 5% on an ARB immediately prior to the onset of the SRC (Table 2). Of these, 16 of the 66 (24%) with hypertensive crisis and 5 of the 10 (50%) with normotensive crisis were on an ACE inhibitor or an ARB prior to the onset of SRC. Over 50% of the patients were also on glucocorticoids immediately prior to the onset of SRC, at a mean dose of 17 mg/d of prednisone (or its equivalents). 

Of the 66 patients classified as hypertensive, 64 satisfied the proposed criteria for hypertensive SRC mentioned in Box 1. Of the 10 patients classified as normotensive, 4 satisfied the proposed criteria for normotensive SRC mentioned in Box 1.

To date, we have collected one-year follow-up data on approximately 1/3 of the cohort. Of these, over 50% have died or remained on dialysis after one year. Collection of one-year follow-up data on the remainder of the patients is ongoing.

## 4. Discussion

Whether ACE inhibitors are associated with a worse prognosis for patients with SRC is an important clinical question, in particular given the widespread availability of these drugs and their perceived benefits in reducing the incidence of SRC. However, given the rarity of SRC, designing a prospective study to address this question is not without considerable logistical problems. Using an international, web-based cohort study design, we identified 76 incident SRC cases over approximately 15 months. We thus believe that this is a feasible method of recruiting a substantial number of patients to study this infrequent vascular manifestation of SSc. 

We had made several assumptions to compute our desired sample size, including that approximately 25% of subjects would be exposed to ACE inhibitors and that the rate of death and/or dialysis after one year would approach 50%. The numbers presented in this preliminary analysis support these assumptions. Thus, after approximately one more year of follow-up, we should have sufficient power to address the important clinical question of the prognosis of patients who develop SRC while on ACE inhibitors compared to those not on these medications.

In order to maximize enrollment for this study, we compiled an extensive list of 589 physicians from around the world with an apparent interest in SSc (i.e., identified from well-established SSc research groups and through international SSc networks), we designed a simple survey requiring less than 5 minutes to complete and we sent out the survey every 2 weeks so as to increase the possibility that the recruiting physician would have easy access to the clinical data. We were especially careful in including incident cases. Indeed, many physicians contacted us to enquire whether they could enroll patients who had had their SRC in the past and were being seen in follow-up. Unfortunately, those patients were not eligible because, having survived their SRC sufficiently long, these prevalent cases were in fact “survivors” and including them could have biased our results.

We encountered several problems in the course of the study. The most important one was that there was no gold standard to define SRC. Given that the recruiting physicians were identified through SSc research groups and had an apparent interest in SSc, we chose to rely on their “expert” opinion. Moreover, we also collected data on the signs and symptoms that they relied on to make their diagnosis. In this preliminary analysis, most patients satisfied the proposed criteria for hypertensive SRC (Box 1). However, additional work will be needed to validate a more sensitive definition of normotensive SRC. 

Another issue that arose was that of ethics approval. The study had been approved by the principal investigators' ethics committee, the data collected for the purposes of this study were obtained through chart review by a treating physician, no direct patient contact was required, and patients were identified using depersonalized study codes. Nevertheless, some recruiting physicians preferred to obtain ethics approval from their local ethics committees. Unfortunately, this imposed a certain workload on them and we did not have funds to support them in this regard. Although this may have delayed initial recruitment in those centers, many participants nonetheless pursued ethics approval presumably based on enthusiasm for the project. 

Thirdly, although the tool that we used to create the survey was very user-friendly, inexpensive, and allowed us to integrate important data quality checks, it allows only basic data analysis. More detailed analysis will require time-consuming data manipulation to transfer the data into more sophisticated programs. Alternative data acquisition formats are available and could be considered for future studies involving more complicated analyses.

Finally, we have had to invest a lot of effort in obtaining one-year follow-up data. The follow-up case report form is somewhat longer and requires approximately twenty minutes to complete. A central research assistant has had to work diligently to encourage recruiting physicians to complete these forms. Contacting them personally by telephone has resulted in improved follow-up data collection. We did not have funds to pay for local research assistants to fill the follow-up forms. Whether this could also have contributed to more efficient collection of follow-up data thus remains unknown. 

Our study will be unable to answer another very important question; that is, whether ACE inhibitors are associated with a reduction in the incidence of SRC. That study would require following patients with mostly early SSc, some exposed and others unexposed to ACE inhibitors, until the occurrence of SRC. Since SRC is infrequent, the sample size for such a cohort study exceeds 1000. Nevertheless, that study using our current design could be feasible. Recruitment would most likely have to occur over several years and strategies to maintain interest in recruitment would have to be developed. Careful collection of follow up data would also be necessary. On the other hand, the costs of maintaining an ongoing web-based survey are really quite minimal. 

This study has some limitations. First, our response rate remains largely uncertain. When we sent the biweekly e-mails, we asked the participants to answer whether or not they had seen a case of SRC in the past two weeks, and if so, go on to fill out the survey. Unfortunately, many participants did not respond to the biweekly e-mails. Thus, it is difficult to know whether they indeed had not seen a case or whether they were not participating (during that particular time period). It is possible that some cases were seen but not entered into the survey, and it is conceivable that their disease characteristics may have been different from those of the cases included in the survey (e.g., some may have had worse and others milder disease). Thus, the response rate and the effect of a nonresponse bias in this study are uncertain. Second, patients who did not have access to a participating physician or those with subclinical disease (e.g., normotensive SRC) whose SRC may have been overlooked by a physician were not captured in this survey. Thus, our results are generalizable to patients diagnosed with SRC and entered into this survey by a participating physician. On the other hand, every two weeks we contacted well over 550 participants identified as members of well-established scleroderma research groups or colleagues of such groups from around the world. SRC is a serious complication of SSc and we thus believe that many if not most SRC cases were, at some point, brought to the attention of one of these perceived SSc experts. 

In conclusion, using an international, web-based prospective cohort design, we identified 76 incident of SRC cases over approximately 15 months. Twenty-two percent (22%) of them were on an ACE inhibitor immediately prior to the onset of their SRC. Follow-up data collection to determine rates of death and/or dialysis after one year according to exposure to ACE inhibitor prior to SRC onset is ongoing. The methodology used for this study is innovative and emphasizes that interinstitutional and international collaboration can contribute significantly to the study of infrequent vascular manifestations of SSc. The ultimate success of this study will depend largely on the goodwill of the recruiting physicians who will have to invest additional time and effort in collecting and providing us with the most complete follow-up data possible. Their dedication will hopefully allow us to answer one of the most pressing ongoing questions related to SRC in the near future. 

## Figures and Tables

**Box 1:**
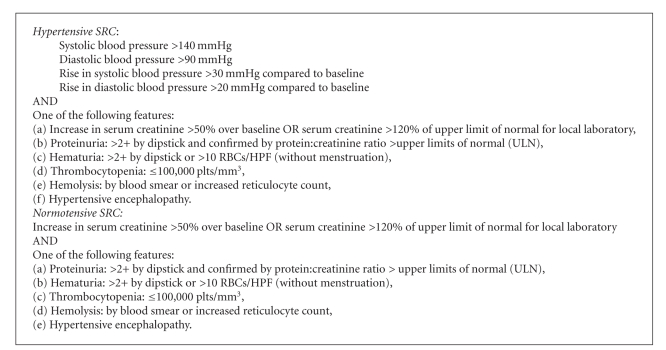
Proposed characteristics of SRC.

**Table 1:** Baseline characteristics of cohort (*N* = 76).

Mean (SD) age, years (SD)	53.3 (12.4)
Women, *N* (%)	52 (68.4)

Ethnic groups, *N* (%)	
White	55 (72.4)
Black	12 (15.8)
Asian	4 (5.3)
Hispanic	3 (4.0)
Native American	2 (2.6)

Disease subsets, *N* (%)	
Diffuse disease	52 (68.4)
Limited disease	19 (25.0)
Sine scleroderma	5 (6.6)

Disease duration (since first non-Raynaud's symptom)	
Median disease duration, years (IQR)	1.47 (0.87, 4.21)
Number (%) with disease duration < 1 year	24 (32)

Countries of origin of study subjects, *N*	
Australia	2
Belgium	1
Brazil	1
Canada	13
Denmark	2
Dominican Republic	1
France	2
Germany	2
Ghana	1
Greece	1
Haiti	2
Hungary	3
Israel	1
Italy	2
Korea	1
Norway	3
Pakistan	3
Poland	1
Spain	2
Switzerland	2
The Netherlands	1
Turkey	3
USA	26

**Table 2:** Characteristics of study patients (*N* = 76).

	*N* (%)
Hypertensive SRC	66 (86.8)
Normotensive SRC	10 (13.2)
ACE inhibitor immediately prior to SRC onset	17 (22.4)
ARB immediately prior to SRC onset	4 (5.3)
Glucocorticoids immediately prior to SRC onset	39 (51.3)
Mean prednisone dose in prednisone equivalents	16.7 mg/day
Nonsteroidal anti-inflammatory drugs immediately prior to SRC onset	9 (11.8)
Cyclosporine immediately prior to SRC onset	1 (1.3)

SRC: scleroderma renal crisis, ACE: angiotensin converting enzyme, and ARB: angiotensin receptor blocker.

**Table 3:** Signs and symptoms of SRC.

Patients with hypertensive SRC (*N* = 66)	*N* (%)
Systolic blood pressure >140 mmHg	64 (97)
Diastolic blood pressure > 90 mmHg	54 (82)
Rise in systolic blood pressure >30 mmHg compared to baseline	46 (70)
Rise in diastolic blood pressure >20 mmHg compared to baseline	37 (56)
Increase in serum creatinine >50 % above baseline OR serum creatinine > 120% of upper limit of normal for local laboratory	60 (91)
Proteinuria: >2+ by dipstick and confirmed by protein:creatinine ratio > upper limits of normal	25 (38)
Hematuria: >2+ by dipstick or >10 RBCs/HPF (without menstruation)	18 (27)
Thrombocytopenia: < 100,000 platelets/mm^3^	20 (30)
Hemolysis: by blood smear or increased reticulocyte count	27 (41)
Hypertensive encephalopathy	9 (14)

Patients with normotensive SRC (*N* = 10)	*N* (%)

Systolic blood pressure >140 mmHg	2 (20)
Diastolic blood pressure >90 mmHg	2 (20)
Rise in systolic blood pressure >30 mmHg compared to baseline	3 (30)
Rise in diastolic blood pressure >20 mmHg compared to baseline	2 (20)
Increase in serum creatinine >50% above baseline OR serum creatinine > 120% of upper limit of normal for local laboratory	9 (90)
Proteinuria: >2+ by dipstick and confirmed by protein:creatinine ratio > upper limits of normal	5 (50)
Hematuria: >2+ by dipstick or >10 RBCs/HPF (without menstruation)	4 (40)
Thrombocytopenia: <100,000 platelets/mm^3^	2 (20)
Hemolysis: by blood smear or increased reticulocyte count	3 (30)
Hypertensive encephalopathy	1 (10)
